# Supplementary material for: The Systems Biology Research Tool: evolvable open-source software
Source: BMC Syst Biol. 2008 Jun 29;2:55. doi: 10.1186/1752-0509-2-55 (PMC2446383; doi:10.1186/1752-0509-2-55)
Supplement: Additional file 1 — SBRT Archive. An archive of the current version of the Systems Biology Research Tool. [file 1752-0509-2-55-S1.zip › sbrt-1.4.0/doc/developers_guide/api/sbrt/shell/text/SimpleFormat.html]

SimpleFormat


|  |  |  |
| --- | --- | --- |
| **Overview** \| **Package** \| **Class** \| **Use** \| **Tree** \| **Deprecated** \| **Index** \| **Help** | |  |
| **PREV CLASS**   **NEXT CLASS** | **FRAMES**    **NO FRAMES**     **All Classes** |  |
| SUMMARY: NESTED \| FIELD \| CONSTR \| METHOD | DETAIL: FIELD \| CONSTR \| METHOD |  |


---


## sbrt.shell.text Interface SimpleFormat<T>

**Type Parameters:**: `T` - the type of object this format applies to.

**All Superinterfaces:**: Format, Formatter<T>, Parser<T>

**All Known Subinterfaces:**: BooleanFormat, CatalystFormat, CatalystListFormat, CatalystSetFormat, ChemSpeciesFormat, CoeffFormat, CollectionFormat<E>, DirEdgeFormat<N>, DoubleFormat, IndexPairFormat<R,C>, IntegerFormat, IntervalFormat, LinearCombFormat<V>, LinearCombTermFormat<V>, LongFormat, MathExprFormat<E,V>, MatrixElementFormat<R,C,E>, PercentFormat, RxnNameExprFormat<E,S>, RxnNameFormat<T>, RxnNameListFormat, RxnNameOrExprFormat, RxnNameSetFormat

**All Known Implementing Classes:**: BoolRelFormatV1, BoundedIntervalFormatV1, CatalystFormatV1, CatalystFormatV2, CatalystListFormatV1, CatalystSetFormatV1, CatalystVerifier, ChemSpeciesFormatV1, ChemSpeciesVerifier, CoeffFormatV1, CoeffFormatV2, DirEdgeFormatV1, DoubleFormatV1, DoubleFormatV2, FileFormatFormat, IndexPairFormatV1, IntegerFormatV1, IntervalFormatV1, IrrevRxnFormatV1, IrrevRxnFormatV1, IrrevRxnFormatV2, LinCombTermFormatV1, LinCombTermFormatV2, LinearCombFormatV1, LinearCombFormatV2, LongFormatV1, MatrixElementFormatV1, NonEmptyStringFormat, PercentFormatVersion1, RevRxnFormatV1, RevRxnFormatV2, RxnNameExprFormatV1, RxnNameFormatV1, RxnNameListFormatV1, RxnNameOrExprFormatV1, RxnNameSetFormatV1, RxnNameVerifier, SenseFormat, SimpleListFormat, SimpleSetFormat, StringFormat, TrueFalseFormat, YesNoFormat

---

``` public interface SimpleFormat<T> extends Format, Formatter<T>, Parser<T> ```

This interface is used to represent simple formats.
A simple format is used to "convert" a single formatted
string into a single object, and vice-versa. This interface
is very similar to the abstract Java class
`java.text.Format` but with fewer implementation
restrictions.

The methods `format(Object)` and `parse(String)`
are inverse functions in the mathematical sense. For
an object `obj` and format `f`, the statement
`f.parse(f.format(obj)).equals(obj)`
must return `true`.

**Author:**
:   This interface was written and documented by
    Jeremiah Wright while in the Wagner lab.

---

**Method Summary**

**Methods inherited from interface sbrt.shell.text.Formatter**

`format`

**Methods inherited from interface sbrt.shell.text.Parser**

`parse`

---


|  |  |  |
| --- | --- | --- |
| **Overview** \| **Package** \| **Class** \| **Use** \| **Tree** \| **Deprecated** \| **Index** \| **Help** | |  |
| **PREV CLASS**   **NEXT CLASS** | **FRAMES**    **NO FRAMES**     **All Classes** |  |
| SUMMARY: NESTED \| FIELD \| CONSTR \| METHOD | DETAIL: FIELD \| CONSTR \| METHOD |  |


---
